# Incidence of orthodontic brackets detachment during orthodontic treatment: A systematic review

**DOI:** 10.12669/pjms.343.15012

**Published:** 2018

**Authors:** Naif Almosa, Hamayun Zafar

**Affiliations:** 1 Dr. Naif Almosa, BDS, NSBO, PhD. Department of Pediatric Dentistry and Orthodontics, College of Dentistry, King Saud University, Riyadh, Saudi Arabia; 2 Dr. Hamayun Zafar, PT, PhD. Department of Rehabilitation Sciences, College of Applied Medical Sciences and Rehabilitation Research Chair, King Saud University, Riyadh, Saudi Arabia. Department of Odontology, Clinical Oral Physiology, Faculty of Medicine, Umea University, Sweden

**Keywords:** Orthodontic treatment, Brackets detachment, Bracket de-bonding, Bracket failure, Prevalence, Incidence

## Abstract

**Objectives:**

To evaluate the incidence of orthodontic brackets detachment during orthodontic treatment.

**Methods:**

Using electronic databases; eligible studies up to January 2018 were retrieved, independently reviewed, and screened. The Coleman Methodology Scoring System (CMS) and Cochrane Collaboration’s tool were used to assess quality and risk of bias in the included studies.

**Results:**

Of the seventeen studies included in the final synthesis, thirteen were categorized as randomized clinical trials (RCTs), one prospective cohort and retrospective survey each, whereas two studies could not be categorized. The number of patients in the selected studies ranged between 19 and 153; the mean age was between 10.5 to 38.7 years, and male to female ratio was 353:495. Almost all studies had a high risk of bias, and more than half of the studies had CMS score of 70 or above. The numbers of brackets examined in the studies ranged between 361 and 3336. The incidence of brackets detachment ranged from 0.6 to 28.3%.

**Conclusions:**

The incidence of brackets detachment during orthodontic treatment is high.

## INTRODUCTION

Orthodontic treatment enhances patients’ physical appearance by correcting malocclusion of teeth. The treatment also improves oral health conditions that are related to malocclusions. These conditions include, mastication difficulties with potential to cause digestion problems, speech impairments, abnormal loading of temporomandibular joints that can lead to severe inflammation and pain, headaches or pain in the patients’ face and neck. Orthodontists use various removable and fixed appliances to treat orthodontic problems. The main components of the fixed orthodontic appliances are brackets that are attached to the teeth using different types of adhesives. The movement of teeth depends on the wires and springs attached to these brackets. Therefore, it is of utmost importance that these brackets remain attached to the teeth during the course of orthodontic treatment. However, brackets detachment “debonding” from the teeth remains one of the major concerns during orthodontic treatment with fixed appliances.^1^-^3^ The bracket bonding procedure plays a major role in achieving an optimal outcome during orthodontic corrective procedures, as the required tooth movement relies upon it.^4^ Bracket detachment during corrective procedures may also lead to increased treatment duration, damage to tooth enamel, and increased chairside-time due to re-bonding procedure.^2^,^3^ Consequently, it could also raise the costs of the overall orthodontic treatment.^4^

Recent advancements in dental materials and bonding techniques has helped to make orthodontic brackets bonding easier, efficient, predictable, and effective.^6^ Orthodontic bonding technique has changed significantly since it was first used in 1950s.^7^ At present, there are direct and indirect bonding techniques used in orthodontic treatment with fixed appliances.^8^,^9^ However, both the techniques have advantages and disadvantages in relation to bond failure rates.^10^-^12^ Although indirect bonding technique has more advantages in terms of shorter initial bonding appointment, higher degree of precision, and more focused results, yet the majority of the orthodontists prefer the direct bonding technique to avoid laboratory involvement.^13^

Bracket detachment is a major concern during orthodontic treatment with fixed appliances, as it can be irritating and in some instances critical in the overall success of the treatment. Presently, there is a tendency towards bonding brackets on all the teeth for providing full arch orthodontic treatment, thus making bracket detachment more critical.^14^-^16^ Previous studies have reported varying incidence of bracket failure following orthodontic brackets bonding.^17^,^18^ Several studies have also compared various techniques of orthodontic bonding and rates of brackets failure.^19^-^27^ However, there are no systematic reviews available on incidence of orthodontic brackets detachment during orthodontic treatment. Therefore, the current study aimed to summarize the evidence regarding the incidence of orthodontic brackets detachment during orthodontic treatment.

## METHODS

### Search Strategies

The electronic databases, PubMed and Web of Science were searched from their inception up to January 2018. Only studies published in the English language were included. The databases were searched using the following keywords: (“Orthodontic treatment” OR “Dental procedures”) AND (“Brackets detachment” OR “Bracket debonding” OR “Bracket bonding” OR “Bracket failure”) AND (“Prevalence” OR “Incidence”). Additionally, the studies were searched manually from the reference lists of the studies identified through databases.

### Study Selection

All the studies investigating brackets detachment during orthodontic treatment with fixed appliances were included. Studies were required to report the incidence of brackets failure as one of the study outcomes.

### Data Extraction

Both authors independently screened the titles and abstracts to exclude irrelevant articles. Full texts of the potential articles were then evaluated to identify eligible studies. Following data were extracted from the included studies: author(s), year of publication, study design, bonding technique used, total number of brackets used, number and incidence of bracket failure, and conclusions. Both authors discussed and reached to an agreement on various items of the collected data.

### Quality Assessment

Both authors evaluated the quality of all the selected studies using the Coleman Methodology Scoring (CMS) system.^28^ The CMS has ten sections with a total of 100 points. Additionally, the Cochrane Collaboration’s tool was used to assess the risk of bias in the included studies. Risk of bias was presented as low, unclear, or high for the each included study.^29^ Both the authors discussed and reached to an agreement on the quality assessment.

### Outcome Measure

The outcome evaluated in this systematic review was the incidence of brackets detachment during orthodontic treatment with fixed appliances.

## RESULTS

### Study Selection

**Fig.1:**
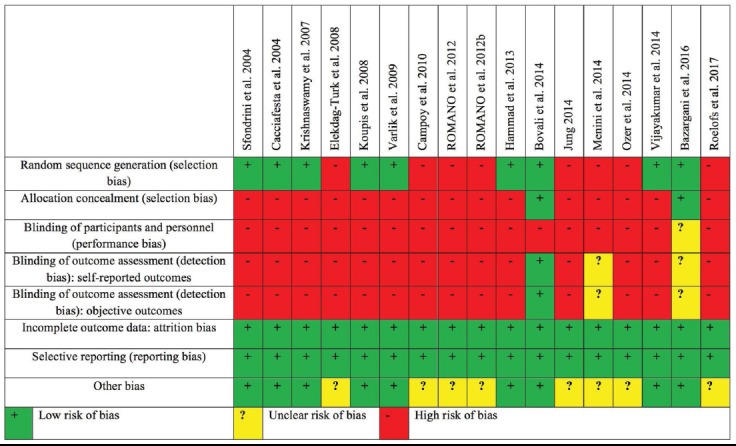
Risk of bias summary: Authors’ judgments about each risk of bias item for each included study.

Based on the titles and abstracts, 222 articles were initially identified. After excluding duplicates and screening the abstracts, 189 studies were not found relevant to objective of this review. Further sixteen articles were excluded due to not matching the inclusion criteria. Therefore, a total of seventeen studies were included in the final synthesis.^1^,^4^,^20^-^24^,^27^,^30^-^38^ The inter-assessor agreement was very good to excellent for initial screening and full-text eligibility (*k =* 0.81 and 0.94 respectively). Figure 1 presents details of study selection process and results of the literature search as per PRISMA guidelines.^24^

### Characteristics of Included Studies

Table I displays the characteristics of all included studies. Among the 17 included studies,^1^,^4^,^20^-^24^,^27^,^30^-^38^ thirteen^20^-^24^,^27^.^30^-^33^,^35^,^37^,^38^ were categorized as RCTs, one prospective cohort,^36^ one retrospective survey,^1^ and two studies did not report about the study design.^4^,^34^ Trials originated from the Netherland,^1^ Brazil,^4^,^34^ India,^20^,^38^ Turkey,^21^,^31^,^37^ Switzerland,^22^ Italy,^23^,^24^,^30^ Sweden,^27^ Greece,^32^ Spain,^33^ Egypt,^35^ and Korea^36^. The number of patients ranged from 19 to 153 with the mean age from 10.5 to 38.7 years. The male to female ratio was 353:495. In most of the included studies, patients were distributed as class I, II, and III malocclusion,^4^,^20^,^23^,^24^,^30^-^32^,^34^,^36^ and stainless steel brackets were used.^20^,^21^,^23^,^24^,^30^,^32^,^33^,^38^ In all the studies, the number of brackets used ranged from 361 to 3336. Four studies compared the halogen light technique with the other adhesive systems on brackets detachment after orthodontic bonding,^20^,^24^,^30^,^32^ while three studies compared direct versus indirect bonding techniques in relation to brackets detachment during orthodontic treatment.^22^,^23^,^38^

**Table I:** Study characteristics and incidence of orthodontic bracket detachment during orthodontic treatment.

Authors	Participants a: Number b: Age, Mean (SD) c: Male/female ratio	Study design	Bracket numbers	Brackets type	Malocclusion class	Adhesive system	Bracket detachment incidence no. (%)	Observation Period (months)	Conclusions
Sfondrini et al. 2004^30^	a: 83 b: 17.3 (4.5) c: 35/48	“split-mouth” with randomization	1434	stainless steel	I, II, III	Halogen light versus plasma arc light	70 (4.9)	12	No significant differences between both techniques.
Cacciafesta et al. 2004^24^	a: 30 b: 16.7 (3.2) c: 12/18	“split-mouth” with randomization	600	stainless steel	I, II, III	Halogen light versus plasma arc light	33 (5.5)	12	As above
Krishnaswamy et al. 2007^20^	a: 30 Age range 12 -20 c: 15/15	“split-mouth” with randomization	544	stainless steel	I, II, III	Light-emitting diode (LED) lamp vs halogen light	41 (7.5)	15	As above
Elekdag-Turk et al. 2008^31^	a: 37 b: 16.5 ? c: 14/23	“split-mouth” with randomization	672	metal	I, II	self-etching primer versus conventional	4 (0.6)	6	Improved bracket survival rate with self-etching primer than the conventional method.
Koupis et al. 2008^32^	a: 37 b: ? c: ?	“split-mouth” with randomization	600	nickel-titanium & stainless steel	I, II, III	Light-emitting diode (LED) lamp vs halogen light	25 (4.20)	9	No significant differences between both techniques.
Varlik et al. 2009^21^	a: 30 Age range 14 -21 c: 14/16	universal numbering system, odd-numbered teeth as control group, even-numbered teeth experimental group.	544	Stainless steel Mini Ovation	?	highly filled light-cured sealant (HFLCS) versus conventional adhesive	18 (3.3)	18	Pro Seal can be used as a preventive measure without affecting the bonding properties of metal brackets.
Campoy et al. 2010^33^	a: 46 b: ? c: ?	prospective controlled clinical trial	531	Stainless steel	?	saliva contamination before bonding versus after bonding	37 (7.1)	6	Either before or after bonding, no significant increase in bracket detachment with saliva contamination
Romano et al. 2012^4^	a: 19 Age range 11-39 c: 7/12	?	380	nickel-titanium	I, II, III	Transbond XT (TXT) composite versus Transbond Plus Color Change (TPCC)	6 (1.6)	6	With both TXT or TPCC methods, a few brackets detached
Romano et al. 2012b^34^	a: 20 Age range 11-15 c: 7/13	?	400	nickel-titanium	I, II, III	Conventional Transbond XT Versus Transbond XT + Transbond Plus Self Etching Primer (TPSEP) adhesive systems Versus Orthodontic Concise and Transbond XT without primer	20 (5)	6	Fewer brackets faliures with conventional Transbond XT and Transbond XT+TPSEP than Orthodontic Concise and Transbond XT without primer.
Hammad et al. 2013^35^	a: 30 b: 14 (?) c: 10/20	“split-mouth” with randomization	538	straight-wire	?	Conventional adhesive vs. Amorphous calcium phosphate-containing adhesive	11 (2.04); 17 (3.1)	6 12	The ACP-containing adhesive seems to be an alternative to conventional adhesives.
Bovali et al. 2014^22^	a: 64 b: 18.5 (4.8) c: 29/35	Randomized controlled trial	?	?	?	Indirect vs direct bonding	17 (28.3)	6	Indirect bonding was statistically significantly faster than direct bonding, Both techniques showed similar risks of failure.
Jung 2014^36^	a: 127 b: 18.6 (6) c: 52/75	prospective cohort study	3061	straight-wire	I, II, III	Molar tubes vs. Anterior brackets	176 (5.7)	12	Bracket detachment rate for molars was greater than anterior teeth.
Menini et al. 2014^23^	a: 52 b: 22.8 (?) c: 25/27	clinical trial	1248	Stainless steel brackets and molar tubes	I, II, III	Indirect vs direct bonding	54 (4.32)	15	No significant differences between both techniques.
Ozer et al. 2014^37^	a: 57 b: 16 (?) c: 18/39	“split-mouth” with alternating quadrants	1140	Self-ligating metal	?	Self-etching primer (SEP) vs. conventional method (CM)	26 (2.57)	22	As above
Vijayakumar et al. 2014^38^	a: 30 b: 21.7 (?) c: 12/18	“split-mouth” with randomization	518	Stainless steel	?	Indirect vs direct bonding	50 (9.6)	6	As above
Bazargani et al. 2016^27^	a: 49 b: 14.4 (1.8) c: 29/20	single-operator, crossmouth, randomized controlled trial (RCT).	908	Metal	?	Primer vs. non-primer	39 (4.2)	18	No difference between both groups, except in younger children the primer setting yielded better results
Roelofs et al. 2017^1^	a: 153 b: 16.6 (10.7) c: 60/93	retrospective survey	3336	Metal and tubes	?	Atropine premedication vs. control	83 (2.5)	18	No significant differences between both techniques.

### Methodological Quality

**Fig.2:**
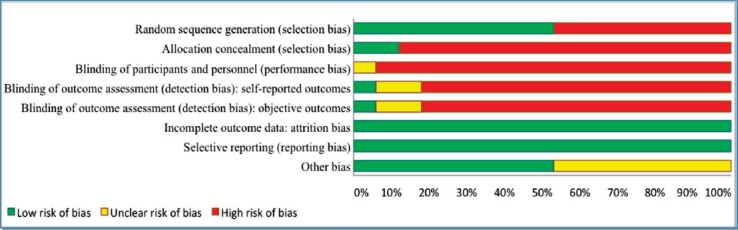
Risks of bias; review authors’ judgments about each risk of bias item presented as percentages across all included studies.

Nine included studies^20^,^22^-^24^,^27^,^30^-^32^,^34^ had CMS score of 70% or above and six studies^4^,^21^-^23^,^33^,^35^,^37^ had CMS score of 60%. Only two studies^1^,^38^ had CMS score of 50%. Two of the studies provided the justification for sample size and provided information about drop outs from the study.^22^,^27^ None of the included studies reported the clinical importance of the results (Table II). Risk of bias is presented as a graph in Figure 2. Almost all the included studies had a high risk of bias,^1^,^4^,^20^-^24^,^30^-^38^ while only one study had an unclear risk of bias.^27^

**Table II:** Methodological quality assessment of included studies based on Coleman Methodology Scoring.^28^

Study	Criteria													

	1	2	3	4	5	6	7	8	9	10	11	12	Score	Scores (%)
Sfondrini et al. 2004^30^	Yes	Yes	Yes	No	N/A	Yes	N/A	Yes	Yes	No	No	Yes	7/10	70
Cacciafesta et al. 2004^24^	Yes	Yes	Yes	No	N/A	Yes	N/A	Yes	Yes	No	No	Yes	7/10	70
Krishnaswamy et al. 2007^20^	Yes	Yes	Yes	No	N/A	Yes	N/A	Yes	Yes	No	No	Yes	7/10	70
Elekdag-Turk et al. 2008^31^	Yes	Yes	Yes	No	N/A	Yes	N/A	Yes	Yes	No	No	Yes	7/10	70
Koupis et al. 2008^32^	Yes	Yes	Yes	No	N/A	Yes	N/A	Yes	Yes	No	No	Yes	7/10	70
Varlik et al. 2009^21^	Yes	Yes	No	No	N/A	Yes	N/A	Yes	Yes	No	No	Yes	6/10	60
Campoy et al. 2010^33^	Yes	Yes	No	No	N/A	Yes	N/A	Yes	Yes	No	No	Yes	6/10	60
ROMANO et al. 2012^4^	Yes	No	Yes	No	N/A	Yes	N/A	Yes	Yes	No	No	Yes	6/10	60
ROMANO et al. 2012b^34^	Yes	Yes	Yes	No	N/A	Yes	N/A	Yes	Yes	No	No	Yes	7/10	70
Hammad et al. 2013^35^	Yes	Yes	No	No	N/A	Yes	N/A	Yes	Yes	No	No	Yes	6/10	60
Bovali et al. 2014^22^	Yes	Yes	No	Yes	N/A	Yes	N/A	Yes	Yes	No	Yes	Yes	8/10	80
Jung 2014^37^	Yes	No	Yes	No	N/A	Yes	N/A	Yes	Yes	No	No	Yes	6/10	60
Menini et al. 2014^23^	Yes	Yes	Yes	No	N/A	Yes	N/A	Yes	Yes	No	No	Yes	7/10	70
Ozer et al. 2014^37^	Yes	Yes	No	No	N/A	Yes	N/A	Yes	Yes	No	No	Yes	6/10	60
Vijayakumar et al. 2014^38^	Yes	No	No	No	N/A	Yes	N/A	Yes	Yes	No	No	Yes	5/10	50
Bazargani et al. 2016^27^	Yes	Yes	Yes	Yes	N/A	Yes	N/A	Yes	Yes	No	Yes	Yes	9/10	90
Roelofs et al. 2017^1^	Yes	No	No	No	N/A	Yes	N/A	Yes	Yes	No	No	Yes	5/10	50

N/A: Not applicable.

### Incidence of Orthodontic Brackets Detachment

The incidence of orthodontic brackets detachment ranged from 0.6 to 28.3% in the selected studies. The follow-up period after bonding of brackets ranged from 6 months to 22 months. The details are provided in Table I. A 6-months detachment incidence was given in seven included studies (0.6% to 28.3%).^4^,^22^,^31^,^33^-^35^,^38^ One study reported 9-months incidence of 4.2%.^34^ Four studies reported 12-months incidence (3.1% to 5.7%).^24^,^30^,^35^,^36^ Two studies reported 15-months incidence (4.3% to 7.5%).^20^,^23^ Three studies reported 18-months incidence (2.5% to 4.2%).^1^,^21^,^27^ Only one study reported a 22-months incidence (2.6%).^37^

## DISCUSSION

As per our knowledge, this is the first systematic review on the incidence of brackets detachment during orthodontic treatment. An increase in incidence of bracket failure is expected with increase in the follow-up period. However, this was not evident from the results of the current review. Only one study reported very high incidence of brackets detachment (28.3%),^22^ while others reported relatively low incident (0.6% to 9.6%).^1^,^4^,^20^,^21^,^23^,^24^,^27^,^30^-^38^ The finding could be attributed to several factors. Firstly, the type of adhesive resin used for bracket bonding could affect the bracket survival. Varlike et al.^21^ concluded that highly filled light-cured sealant can be used as a preventive measure without affecting the bonding properties of metal brackets. Similarly, Romano et al.^4^ reported less number of bracket failure following the application of Transbond XT (TXT) composite or Transbond Plus Color Change (TPCC). Furthermore, Hammad et al.^35^ have recommended using amorphous calcium phosphate-containing adhesive to minimize risk of bracket failure. Secondly, direct and indirect bonding technique could be another reason for different rates of bracket detachment during orthodontic treatment. Indirect bonding technique is significantly faster than direct bonding, however, both techniques have shown similar risks of brackets bonding failure^22^,^23^,^38^

Out of the seventeen studies included in this review, eight studies^1^,^4^,^21^,^33^,^35^-^38^ had low CMS score (≤ 60%), which indicates low methodological quality. Various items were not met by most of the included studies, therefore, future studies investigating incidence of brackets detachment after orthodontic treatment considering these items are recommended. The lack of information about the sample size estimation and dropouts could limit the validity of the results. Additionally, a lack of information about the patient’s description could also limit the generalizability of results.

Of the seventeen studies included in this review, almost all the included studies had a high risk of bias,^1^,^4^,^20^-^24^,^30^-^38^ while only one study had an unclear risk of bias.^27^ Several items including allocation concealment and blinding of participants, personnel and outcome assessor were not met by most of the included studies. A previous study has reported the importance of blinding to reduce the performance and detection bias.^39^

Limitations: It was heterogeneity among the studies as related to patients’ selection criteria, treatment techniques, outcome criteria, and length of follow-up, indicating lack of sufficient body of literature available on this topic. The present review did not assess the factors associated with brackets detachment during orthodontic treatment. Nevertheless, the present review has provided new evidence-based information on incidence of bracket failure during orthodontic treatment. Orthodontists need to adopt all the possible measures to prevent bracket failure during treatment with fixed orthodontic appliances.

## CONCLUSIONS

The present review indicates a high incidence of brackets detachment during orthodontic treatment. However, more high quality studies with larger samples are recommended to improve the evidence on the prevalence and incidence of brackets detachment during orthodontic treatment.

### Authors’ Contribution

**NA:** Conceiving the research idea, literature search, categorization of included studies, data analyses, data interpretation, manuscript preparation and editing.

**HZ:** Literature search, categorization of searched studies, data analysis and interpretation, manuscript writing.
